# Verification of radiodynamic therapy by medical linear accelerator using a mouse melanoma tumor model

**DOI:** 10.1038/s41598-018-21152-z

**Published:** 2018-02-09

**Authors:** Junko Takahashi, Mami Murakami, Takashi Mori, Hitoshi Iwahashi

**Affiliations:** 1 Biomedical Research Institute, National Institute of Advanced Industrial Science and Technology (AIST), Tsukuba, Ibaraki Japan; 2 Faculty of Applied Biological Sciences, Gifu University, Gifu, Japan

## Abstract

Combined treatment with 5-aminolevulinic acid (5-ALA) and X-rays improves tumor suppression *in vivo*. This is because the accumulated protoporphyrin IX from 5-ALA enhances the generation of ROS by the X-ray irradiation. In the present study, a high-energy medical linear accelerator was used instead of a non-medical low energy X-ray irradiator, which had been previously used. Tumor-bearing mice implanted with B16-BL6 melanoma cells were treated with fractionated doses of irradiation (in total, 20 or 30 Gy), using two types of X-ray irradiator after 5-ALA administration. Suppression of tumor growth was enhanced with X-ray irradiation in combination with 5-ALA treatment compared with X-ray treatment alone, using both medical and non-medical X-ray irradiators. 5-ALA has been used clinically for photodynamic therapy. Thus, “radiodynamic therapy”, using radiation from medical linacs as a physical driving force, rather than the light used in photodynamic therapy, may have potential clinical applications.

## Introduction

For cancer treatment, radiotherapy is preferred to surgical resection because it is non-invasive, and preserves organ structure and function. However, acute toxicity and potential long-term adverse effects often limit the dose of radiation to levels that are insufficient for controlling tumors, particularly when a tumor resides in close proximity to a radiosensitive organ. Use of tumor-selective agents that enhance radiation effects in tumors, but spare normal tissue, can improve therapeutic efficacy, by ameliorating local control of the tumor without increasing the radiation dose. Some possible radiosensitizers induce apoptosis by inhibiting nucleic-acid synthesis, angiogenesis, DNA repair, and cell signaling^1,2^. Others affect cellular redox stress^3,4^. To date, no radiosensitizers with clinical benefits have been discovered.

We reasoned that the effects of radiation might be enhanced by amplifying the radiation dose by physico-chemical reaction, because it is easy to control the radiation dose. Organic scintillators are materials that exhibit scintillation, the property of luminescence, when excited by ionizing radiation during physicochemical reactions. The most common scintillators are anthracene, stilbene, and naphthalene^5^; however, these can be toxic to cells. Much less work has been done on the use of the physicochemical reactions of organic materials under ionizing radiation for radiosensitization.

Previously, we investigated protoporphyrin IX (PpIX) as a candidate oncotropic radiosensitizer. Specifically, we measured the types and amount of reactive oxygen spices (ROS) generated by X-ray irradiation using ROS detection reagents, with ethanol as a quencher of ROS in solutions containing different concentrations of PpIX. We estimated the amount of the major ROS—the hydroxyl radical (∙OH), superoxide anion (O_2_^∙−^), and singlet oxygen (^1^O_2_)—resulting from PpIX treatment^6^. Systemic application of 5-aminolevulinic acid (5-ALA) leads to selective accumulation of PpIX in tumor cells. Subsequent excitation with light forces PpIX into its singlet state, which emits fluorescence upon returning to the ground state, and producing ROS^7,8^. 5-ALA has been used clinically for fluorescence-guided surgery and photodynamic therapy (PDT)^9,10^. Our findings suggest that it is possible to use X-rays as an energy source instead of light, as used in PDT.

5-ALA pre-treatment increases the efficacy of radiotherapy with multi-dose ionizing radiation with 100–160 kV X-rays in animal cancer models^11,12^. The most commonly used radiotherapy device in practice today is the linear accelerator (linac), and its therapeutic energy range is above 1 MeV. In the present study, we evaluated two kinds of radiotherapy devices with different energy outputs: the linear accelerator with 4 MeV beam energy, and the 160 kV nominal X-ray tube. We also evaluated the impact of irradiation dose for tumor suppression in combined treatment with 5-ALA *in vivo*.

## Materials and Methods

### Cell culture

The B16-BL6 mouse melanoma cell line was supplied by the Riken Cell Bank (Tsukuba, Japan), and was cultured in RPMI1640 containing 10% FBS (Wako chemicals, Osaka, Japan) in a 5% CO_2_ humidified incubator at 37 °C.

### Animal studies

Generation of the B16-BL6 mouse melanoma model was described previously^13,14^. Mice were randomized into 4 groups (n = 4 or 5 per group) after implantation of B16-BL6 cells as follows: (1) no treatment (NT), (2) 5-ALA (Wako chemicals, Osaka, Japan) treatment (ALAT), (3) X-ray treatment for a total dose of 20 Gy (20XT), (4) 5-ALA and X-ray treatment for a total dose of 20 Gy (ALA-20XT), (5) X-ray treatment for a total dose of 30 Gy (30XT), and (6) 5-ALA and X-ray treatment for a total dose of 30 Gy (ALA-30XT). Beginning at three days after cell injection, mice in the 20XT and ALA-20XT groups were irradiated with 2 Gy daily (quaque die, q.d.) × 5 days/week × 2 weeks, making the total dose of irradiation received 20 Gy. Mice in the 30XT and ALA-30XT groups were irradiated with 3 Gy daily × 5 days/week × 2 weeks, making the total 30 Gy. Mice in the ALAT, ALA-20XT, and ALA-30XT groups were administered 5-ALA diluted in PBS locally at a concentration of 50 mg/kg 4–5 hours before X-ray irradiation. The mice in the NT, 20XT, and 30XT group received PBS at the same time. Tumor volume, based on caliper measurements, was calculated every day according to the following formula: tumor volume = shortest diameter^2^ × largest diameter × 0.5. At 2–4 h after the last 10 sessions of X-ray irradiation, the mice were sacrificed. The tumors were weighed using an electronic balance, then submerged in RNA stabilization solution (RNAlater, Sigma-Aldrich Inc., St. Louis, MO, USA) in 2 mL tubes, incubated at 4 °C overnight, and stored at −80 °C. Histologic sections fixed in 10% formalin neutral buffer solution were stained with hematoxylin and eosin. RNA in histologic sections was digested with RNase A (0.5 µg/mL) for 15 minutes at room temperature before propidium iodide (2.5 µg/mL) staining to visualize DNA on a Carl Zeiss LSM Pascal confocal microscope (Carl Zeiss, Tokyo, Japan).

### X-ray irradiation

Irradiation was carried out in a linear accelerator (PRIMUS Mid-Energy, Toshiba Medical Systems, Tokyo, Japan) or Faxitron CP-160 irradiator (Faxitron X-ray Corporation), with X-ray energy outputs of 4 MeV and 160 kV, and dose rates of 1.0 Gy/min and 2.5 Gy/min, respectively.

Mice were secured in plastic holders with an opening above the tumor region. The collimated X-ray beam irradiated an area of 24 × 24 mm^2^ at the tumor site, which was large enough to cover the entire area of the largest tumor. During irradiation with the linear accelerator, tissue-equivalent bolus material was placed on the skin to increase the surface dose.

### Microarray analysis

Microarray analysis of tumor samples irradiated with the linear accelerator was performed as previously described^14^. Data were normalized by quantile normalization, and were analyzed using GeneSpring GX software version 10.0.1 (Agilent Technologies, CA, USA). The gene ontology (GO) database (http://www.geneontology.org/) was used to functionally categorize the gene expression profiles. GO terms were obtained from Agilent Technologies eArray (https://earray.chem.agilent.com/earray/).

All microarray experiments conducted were MIAME compliant, and the raw data has been deposited in the gene expression omnibus (GEO) database (accession number GSE GSE92972, https://www.ncbi.nlm.nih.gov/geo/query/acc.cgi?acc=GSE92972).

### Quantitative RT-PCR (qRT-PCR)

Total RNA was subjected to reverse transcription using iScript™ reverse transcription super-mix (Bio-Rad Laboratories, CA, USA), and used for RT-PCR in the 2-step qRT-PCR assays. Gene-specific primers were used to amplify target transcripts using an MJ mini personal thermal cycler (Bio-Rad Laboratories, CA, USA), using Sso Fast Eva Green Supermix (Bio-Rad Laboratories, CA, USA), according to the manufacturer’s instructions. Primer sequences for amplification of mouse *Actb* (ID 6671509a1), mouse *p21* (ID 162287332c1), mouse *Gadd45a* (ID 6681149a1), mouse *Sod2* (ID 31980762a1), and mouse *Gpx7* (Primer Bank ID 13195626a1) were obtained from Primer Bank, a public resource for PCR primers^15^. Relative transcript quantities were calculated using the ΔΔCt method with *Actb* as the reference gene.

### Evaluation of ROS generation *in vivo*

To evaluate ROS formation, we measured luminescent signals produced by coelenterazine, which is used as a probe for O_2_^∙−^ *in vivo*^16,17^. Native or unmodified coelenterazine was purchased from Cyman Chemical (MN, USA). Coleneterazine was dissolved in ethanol and diluted using PBS (final ethanol concentration, <2%). To evaluate the luminescent signals of coelenterazine *in vitro*, PpIX concentrations ranging from 0 to 1 µg/mL were exposed to 1 µM colenterazine, and irradiated (0, 3, 10, or 30 Gy X-ray) in a microplate containing only reagents and no cells. To detect ROS generation *in vivo*, X-ray irradiation was performed after the tumor volume reached approximately 250 mm^3^. The 5-ALA treatment groups were locally administrated 5-ALA at a concentration of 50 mg/kg, about 4 to 5 hours before X-ray irradiation. Mice were anesthetized using isoflurane and administered 0.1 mg/kg native coelenterazine locally, 10 min before subjecting them to 3 Gy irradiation using the Faxitron CP-160 irradiator. Luminescent signals were measured before and after X-ray irradiation using IVIS 100 (Xenogen, CA, USA), which consists of a light-tight chamber equipped with a cooled CCD camera. Data are represented as the total flux (p/s).

### Statistical analysis

Tumor volume, tumor weight, body weight, gene expression based on qRT-PCR or microarray, cell cycle distribution, and chemiluminescent signal produced by coelenterazine were analyzed by one-way factorial ANOVA followed by Tukey-Kramer multiple comparisons test. In cases where the variance was not homogenous, the Games–Howell post hoc test was used. Differences were considered statistically significant at *p* < 0.05. Pearson’s correlation coefficient was calculated to identify correlations in gene expression among individual microarray data sets. Before analysis by these multiple comparisons tests, Fisher’s Z-transform was used to fit the data to a normal distribution. Hierarchical clustering using the Euclidean distance method and Ward’s linkage was also performed on the 22 arrays.

### Ethical considerations

All procedures for the care and treatment of animals were performed according to the Japanese act on the Welfare and Management of Animals, and the Guidelines for the Proper Conduct of Animal Experiments issued by the Science Council of Japan. All experimental protocols were approved by the Committee for the Care and Use of Experimental Animals at the National Institute of Advanced Industrial Science & Technology (AIST) (permit number 2016-097).

## Results

### Combined treatment with 5-ALA and X-ray irradiation

**Figure 1:**
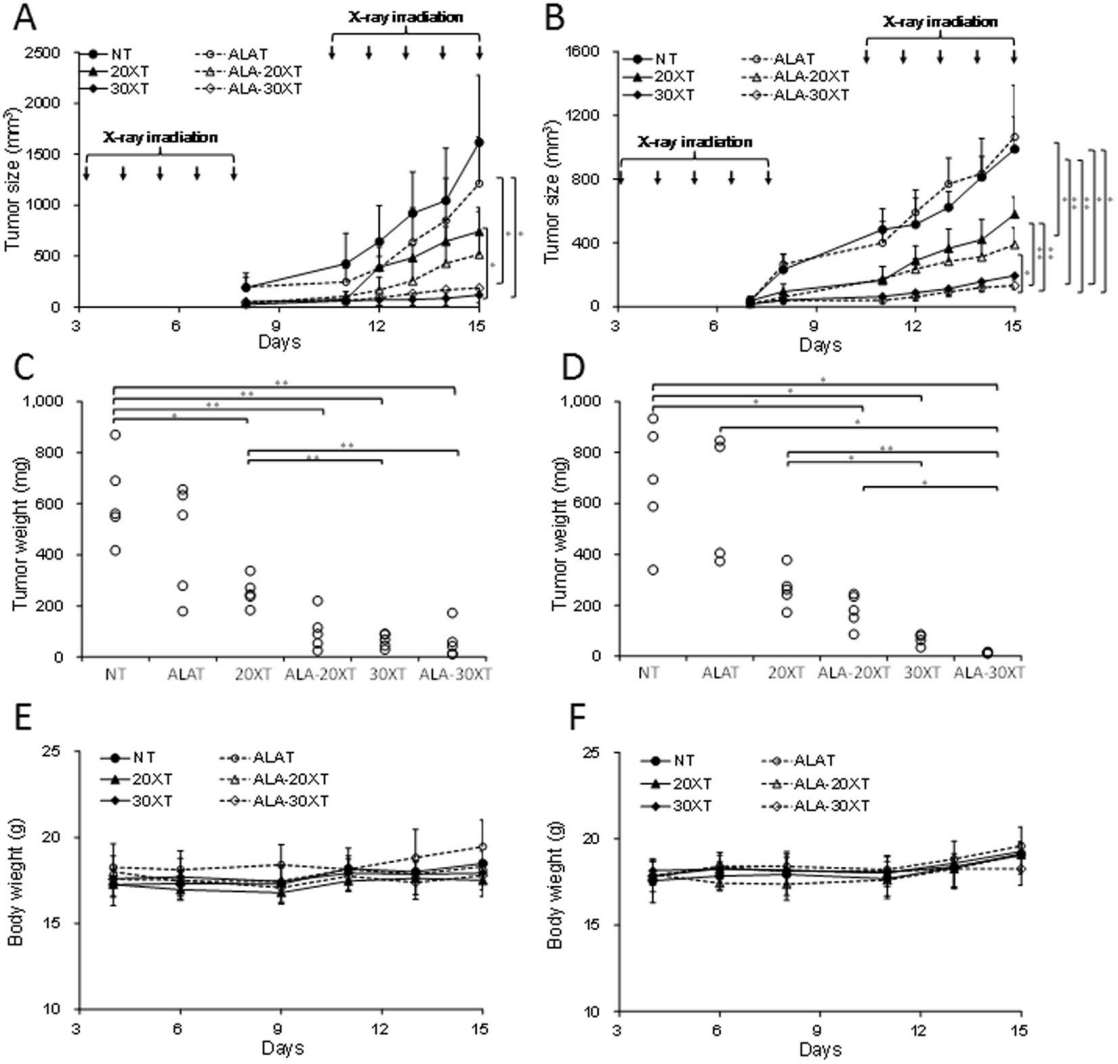
5-ALA potentiates B16-BL6 tumor suppression by X-ray irradiation using two different devices: a linear accelerator with 4 MeV beam energy (**A**,**C**,**E**), and a CP-160 Cabinet X-Radiator™ System (nominal X-ray tube voltage: 160 kV) (**B**,**D**,**F**). C57BL/6J mice with B16-BL6 cells were divided into the following 6 groups: NT, no treatment; ALAT, 5-ALA treatment; 20XT, 2 Gy/day for 10 days; ALA-20XT, 5-ALA treatment followed by 2 Gy/day for 10 days; 30XT, 3 Gy/day for 10 days; ALA-30XT, 5-ALA treatment followed 3 Gy/day for 10 days. Data are shown as means ± SD (n = 4 or 5, *p < 0.05, **p < 0.01). Tumor size was measured after inoculation with B16-BL6 at day 0 (**A**,**B**). Tumors were excised from animals and weighed after 10 sessions of fractionated irradiation (**C**,**D**). Body weights were measured 3 times a week (**E**,**F**).

A C57BL/6J melanoma tumor model was used to evaluate the effect of irradiation dose in combined treatment with 5-ALA on tumor suppression *in vivo*. In the present study, we used two types of X-ray irradiation devices: a linear accelerator with 4 MeV beam energy, and a cabinet type X-ray irradiator with 160 kV nominal X-ray tube voltage. The tumor size decreased with increasing total irradiation dose, and was notably reduced in mice receiving 5-ALA treatment plus a total dose of 20 Gy of irradiation, regardless of X-ray device used (Fig. 1A,B). Tumor weight decreased in a similarly dose-dependent fashion, and was notably reduced in mice receiving 5-ALA plus a total dose of 20 Gy and 30 Gy of irradiation (Fig. 1C,D). The measurement of carefully cleaned tumor weight provided a more precise measurement of tumor growth suppression, as some skin was included in tumor size measurements using calipers. 5-ALA plus irradiation synergistically suppressed B16-BL6 tumor growth at the integral doses of 20 Gy and 30 Gy using both X-ray irradiation devices, without decreasing body weight (Fig. 1E,F).

### Morphological observation of tumor tissues

**Figure 2:**
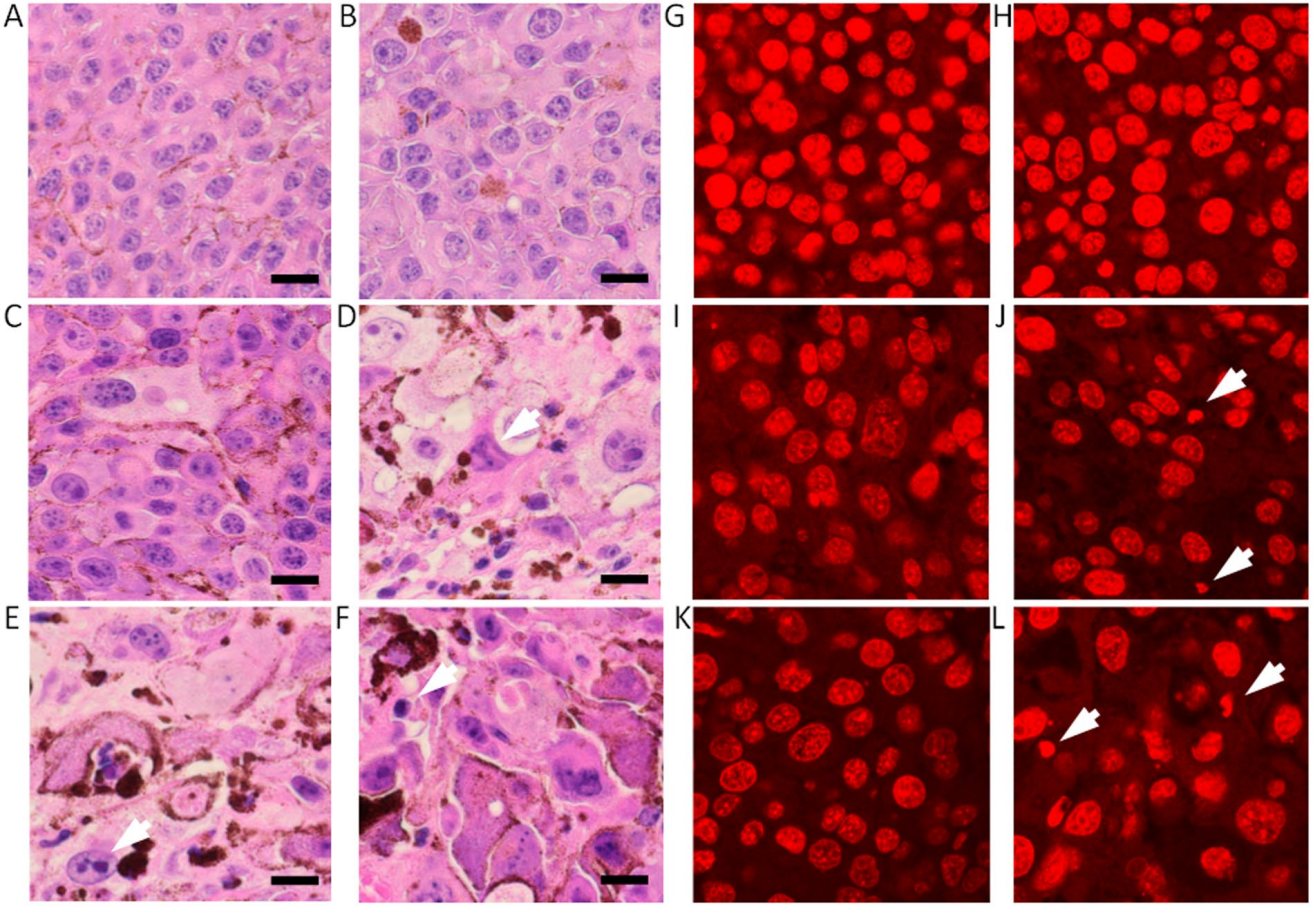
Morphology of a tumor section observed after HE staining (**A**–**F**), and PI staining (**G**–**L**). After 10 fractional dosed of 20 or 30 Gy X-ray irradiation, the mice were sacrificed. Tumors were fixed in formalin solution (neutral buffered, 10%) and stained with HE (**A**) NT, (**B**) ALAT, (**C**) 20XT, (**D**) ALA-20XT, (**E**) 30XT, (**F**) ALA-30XT group tissues. The tumor tissue in NT and ALAT groups showed uniform cells, whereas the ALA-20XT and 30XT group tissues showed more heterogeneous cells with fragmented nuclei (arrowhead). Bars represent 20 μm.

The morphological characteristics of the tumors were obtained by HE and PI staining. The control NT and ALAT tumor tissue cells were fairly uniform (Fig. 2A,B), whereas the X-ray treated tumor cells were not (Fig. 2C–F). X-ray treatment resulted in the formation of giant cells with aberrant nuclear morphologies due to mitotic catastrophe. The NT and ALAT cells had uniform nuclei (Fig. 2G,H), while cells with enlarged or shrunken nuclei were observed in the X-ray treated tumor tissue. Cells with fragmented nuclei, or cells without stained nuclei, were typically observed in the ALA-20XT, 30XT, and ALA-30XT tissue (Fig. 2J–L).

### Analysis of gene expression profiles by microarray

We further characterized the effects of 5-ALA and linac X-ray irradiation on gene expression by microarray. Each sample (NT, ALAT; n = 3 20XT, ALA-20XT, 30XT, ALA-30XT; n = 4) was analyzed using a mouse gene expression microarray consisting of 43,379 oligonucleotide probes.

**Figure 3:**
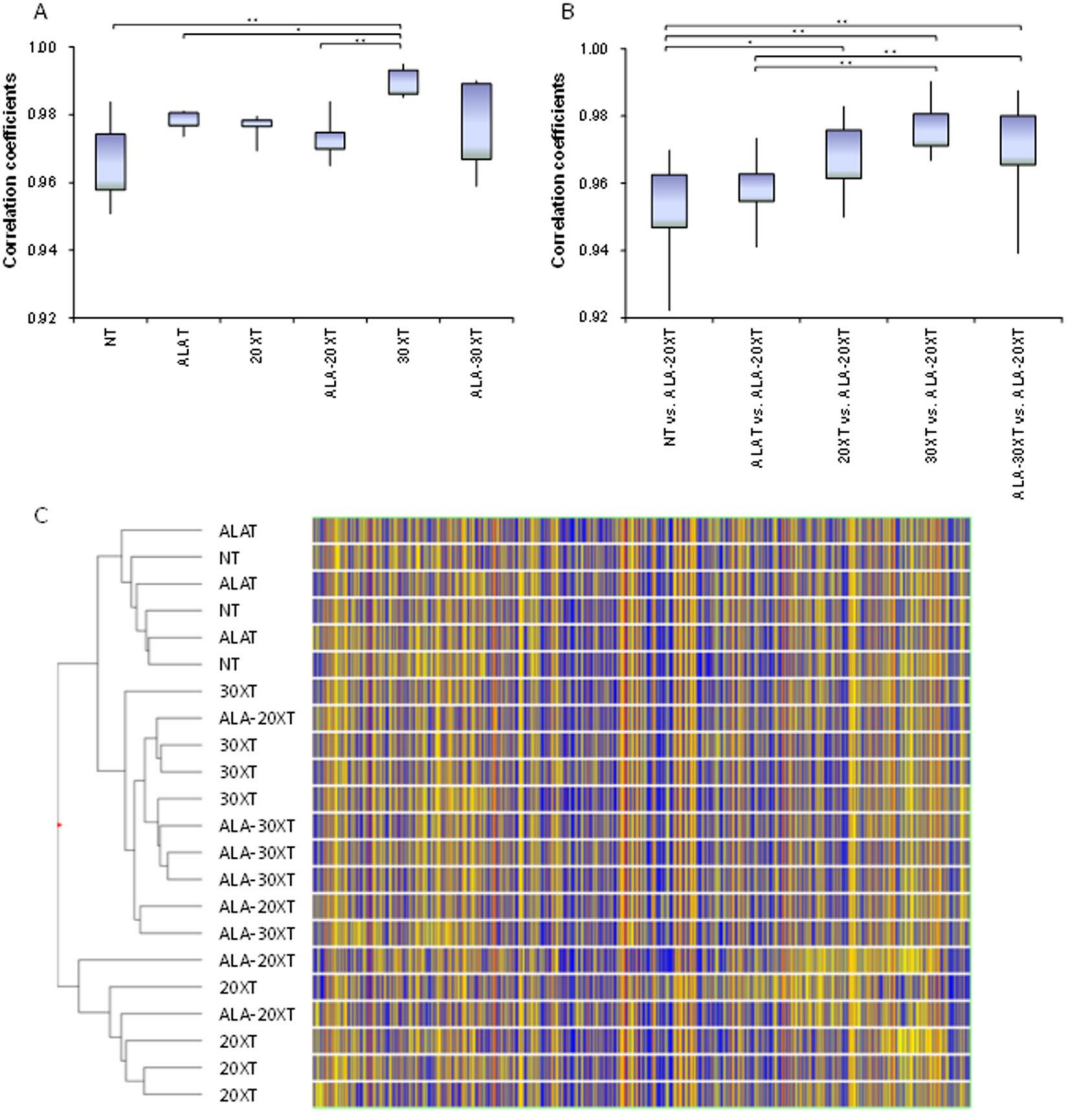
Summary of microarray analysis. (**A**) Correlation coefficients within treatment groups. (**B**) Correlation coefficients between different treatment groups. The mean correlation coefficient between individuals in the NT and ALA-20XT groups is represented as “NT vs. ALA-20XT”. The lower and upper limits of the boxes represent the 25th and 75th percentiles, respectively. The lower and upper whiskers denote the minimum and maximum values, respectively. Fisher’s Z-transformation was used to normalize the correlation distribution, and continuous variables were analyzed using one-way factorial ANOVA followed by the Tukey-Kramer multiple comparisons test. In cases where the variances were not homogenous, a Games–Howell test for multiple groups was performed (*p < 0.01). (**C**) Hierarchical clustering of 22 arrays from 6 groups, with Euclidean similarity measure and Ward’s linkage to visualize the expression profiles of genes between groups. The heat map shows the gene expression for arrays in rows, and the dendrogram representing their similarity. Clustering was performed using normalized signal intensity values for all 22 arrays.

We evaluated the variation in the correlation coefficients of individual samples both within and between treatment groups. Pearson’s correlation coefficient was used for the correlation analysis. The mean correlation coefficients for gene expression profiles among individuals within a treatment group are shown in Fig. 3A, and reflect the individual differences in gene expression. The correlation coefficients of the gene expression in tumors in the 30XT group were higher than in other treatment groups, which is consistent with reduction in tumor weight variation observed in this group compared to that of the other groups. We also analyzed the expression profile correlation between the different treatment groups vs. that of ALA-20XT (Fig. 3B). The differences in correlation coefficient between the groups without X-ray treatment and the groups with X-ray treatment were statistically significant. The differences in correlation coefficient between 30XT vs. ALA-20XT, or ALA-30XT vs. ALA-20XT, were higher than between 20XT vs. ALA-20XT. This suggests that the gene expression in the ALA-20XT samples was more similar to that in the 30XT or ALA-30XT samples than in the 20XT samples.

Hierarchical clustering was performed on the 22 arrays using the Euclidean distance method and Ward’s linkage. The samples clustered into three clusters (Fig. 3C): one containing the non-irradiated groups (NT and ALAT), a second containing 20XT and half of 2ALA-20XT, and a third containing 30XT, ALA-30XT and half of ALA-20XT. We therefore surmised that the gene expression profile of ALA-20XT was located in the middle of the gene expression profile of 20XT and 30XT/ALA-30XT.

### Functional analysis and functional validation using marker genes

We selected genes with altered expression in each treatment group compared to the non-irradiated groups (NT and ALAT) based on their *p*-values (*p* < 0.01). Based on treatment, these genes were separated into up- and down-regulated groups (Supplementary Table 1). The selected genes were functionally annotated in DAVID. We then extracted GO terms based on the number of genes in each GO category. Supplementary Table 2 shows the differentially represented GO terms in the treated groups vs. the non-irradiated groups (FDR < 0.01). The GO terms associated with the downregulated genes in the treated groups were mostly related to cell cycle, including DNA metabolic processes and RNA metabolic processes. The GO terms associated with the upregulated genes included oxidation-reduction processes. We investigated whether the cell cycle was disrupted by 5-ALA treatment prior to X-ray irradiation *in vitro*. The population in G2/M phase increased with increasing X-ray irradiation doses, and this phenomenon was further enhanced by 5-ALA treatment (Supplementary Figure 1).

**Figure 4:**
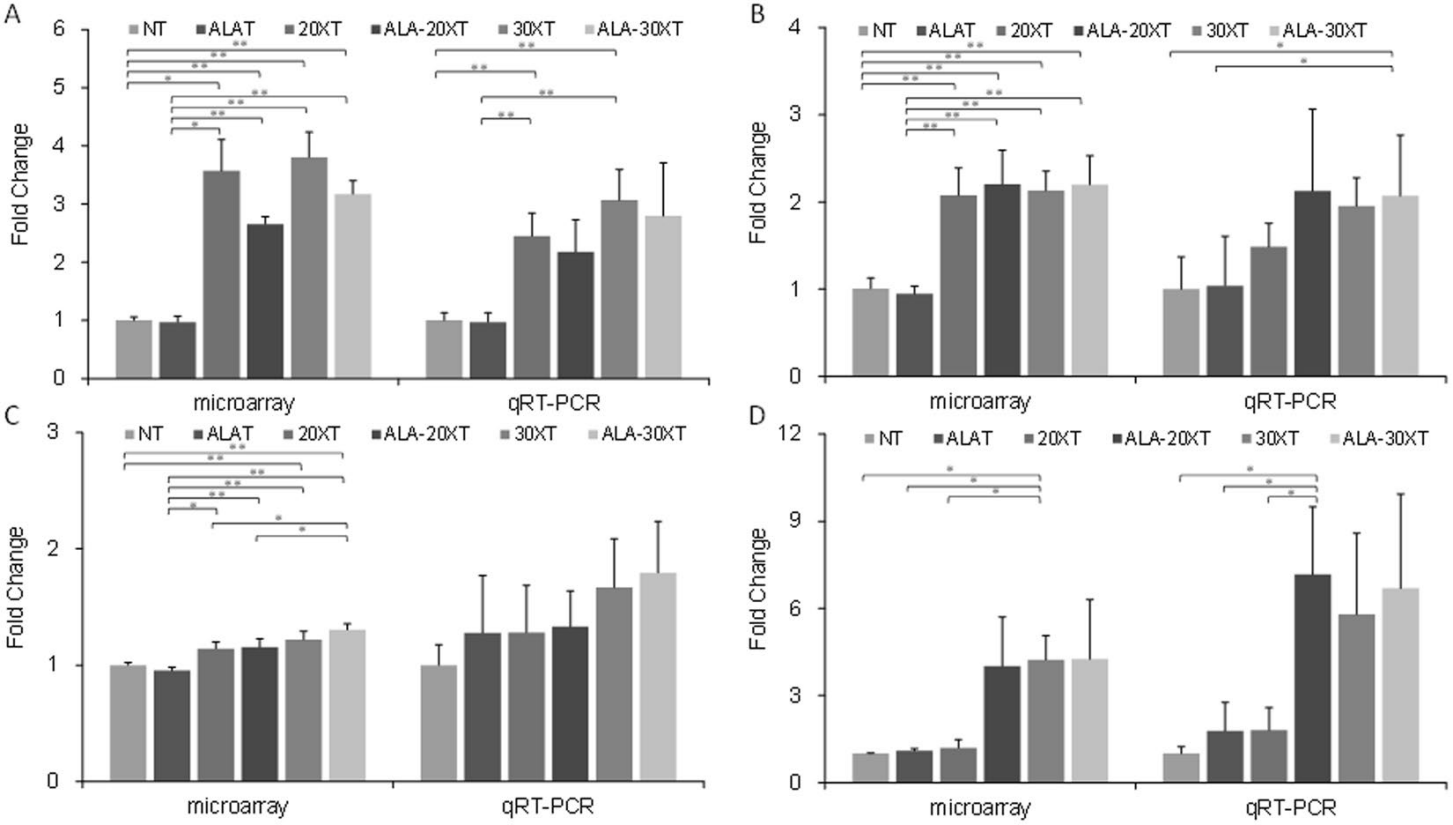
Marker gene expression for cell-cycle regulation: (**A**) *p21*, (**B**)*Gadd45a*. Marker gene expression for oxidative reduction: (**C**) *Sod2*, (**D**) *Gpx7*. Data represents means ± SD (*p < 0.05, **p < 0.01).

Based on the functional analysis results, we examined the expression level of genes that are representative of cell-cycle regulation and oxidation-reduction. We selected *p21* and *Gadd45a* as marker genes representative of cell-cycle regulation, and *Sod2* and *Gpx7* as marker genes representative of oxidation-reduction (Fig. 4A–D). As a whole, the expression changes identified by the microarray analysis were confirmed by the qRT-PCR analysis. The expression of *p21* increased following X-ray irradiation, regardless of irradiation dose or 5-ALA treatment. *Gadd45a* and *Sod2* expression increased with increasing irradiation dose, while *Sod2* expression was further enhanced by 5-ALA treatment. *Gpx*7 was not expressed in cells exposed to 20XT treatment, but was expressed in cells exposed to ALA-20XT, 30XT, and ALA-30XT treatment.

### Luminescent signals produced by coelenterazine detected in response to X-ray irradiation

**Figure 5:**
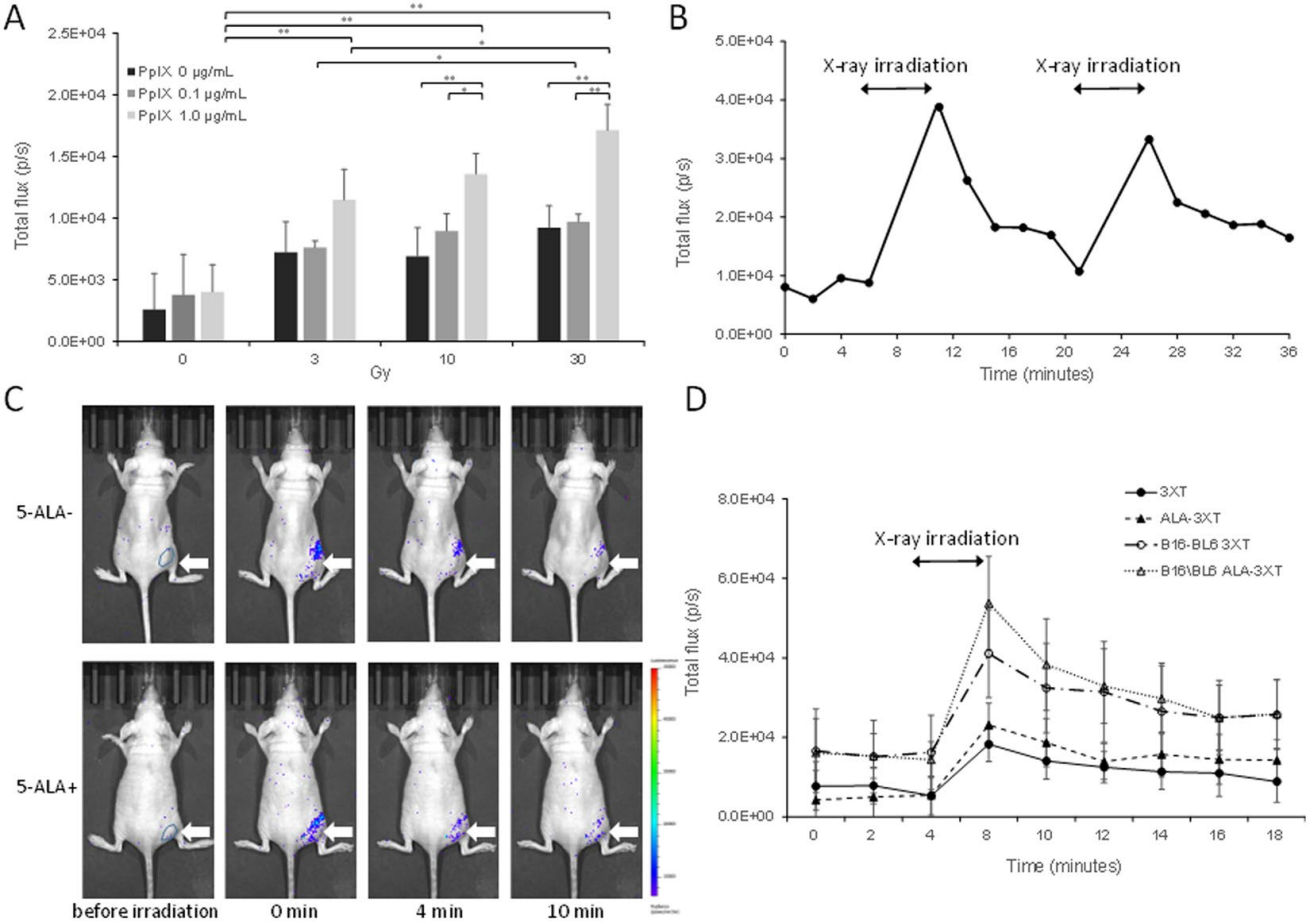
Coelenterazine produces luminescent signals following X-ray irradiation. (**A**) The chemiluminescent signal increases with increasing PpIX concentration and X-ray dose in a microplate containing only reagents and no cells.Data represent the mean ± SD (n = 4, *p < 0.05, **p < 0.01). (**B**) Luminescent signals recorded at different time points after X-ray irradiation. When the irradiation was subsequently repeated, the signal again increased and decayed over time. (**C**) A typical example of *in vivo* imaging of a mouse injected with coelenterazine before and after 3 Gy X-ray irradiation w/o 5-ALA treatment with tumor (arrowhead). (**D**) The average luminescence before and after 3XT, 3 Gy; ALA-3XT, 5-ALA treatment followed by 3 Gy irradiation with or without B16-BL6 tumor. Data represent the mean ± SD (n = 6).

We tried to evaluatedmeasure ROS production in tumor caused by X-ray irradiation. Chemiluminescence could be detected, and was observed to be dependent on the PpIX concentration and X-ray irradiation dose in a microplate containing only reagents and no cells (Fig. 5A). For evaluating ROS generation *in vivo*, we measured the luminescent signals produced by coelenterazine after 3 Gy X-ray irradiation. Immediately after X-ray irradiation, the intensity of luminescence increased and subsequently decreased with time, with or without 5-ALA treatment. When irradiation was subsequently repeated, the signal again increased and decayed over time (Fig. 5B). It was observed to be more conspicuous in tumor treated with 5-ALA (Fig. 5C,D). Since coelenterazine detects superoxide^15,16^, 5-ALA treatment potentially increased superoxide production.

## Discussion

The most common form of radiation used in practice today is the high-energy photon, which is created electronically by devices such as medical linear accelerators. The interaction between photon and tissue differs with photon energy. The Compton effect is the most common clinically occurring interaction, as most radiation treatments are performed at energy levels of about 6–20 MeV^18^. We previously studied the combined effect of 5-ALA and X-ray irradiation using a tube voltage of 100–160 kV, in which the photoelectric effect predominates in tissue^11,14^. In the present study, we evaluated the tumor suppression effects of two kinds of devices that produce different energy: a linear accelerator with 4 MeV beam energy, and a cabinet type X-ray irradiator with 160 kV nominal X-ray tube voltage. The effective energy of the linear accelerator was estimated to be about 2 MeV^19^. The operating dose rate was 2.5 Gy/min. A 0.5 mm thick aluminum plate was used for CP-160, and the dose rate was 1 Gy/min. The X-ray spectrum of this source is broad, with a mean X-ray energy of 75 keV^20^. Although two devices had large differences in their energy outputs, tumor suppression improved similarly in animals treated by both (Fig. 1). A combined treatment with 5-ALA seems to improve the effect, for the dose rates used in this study.

**Figure 6:**
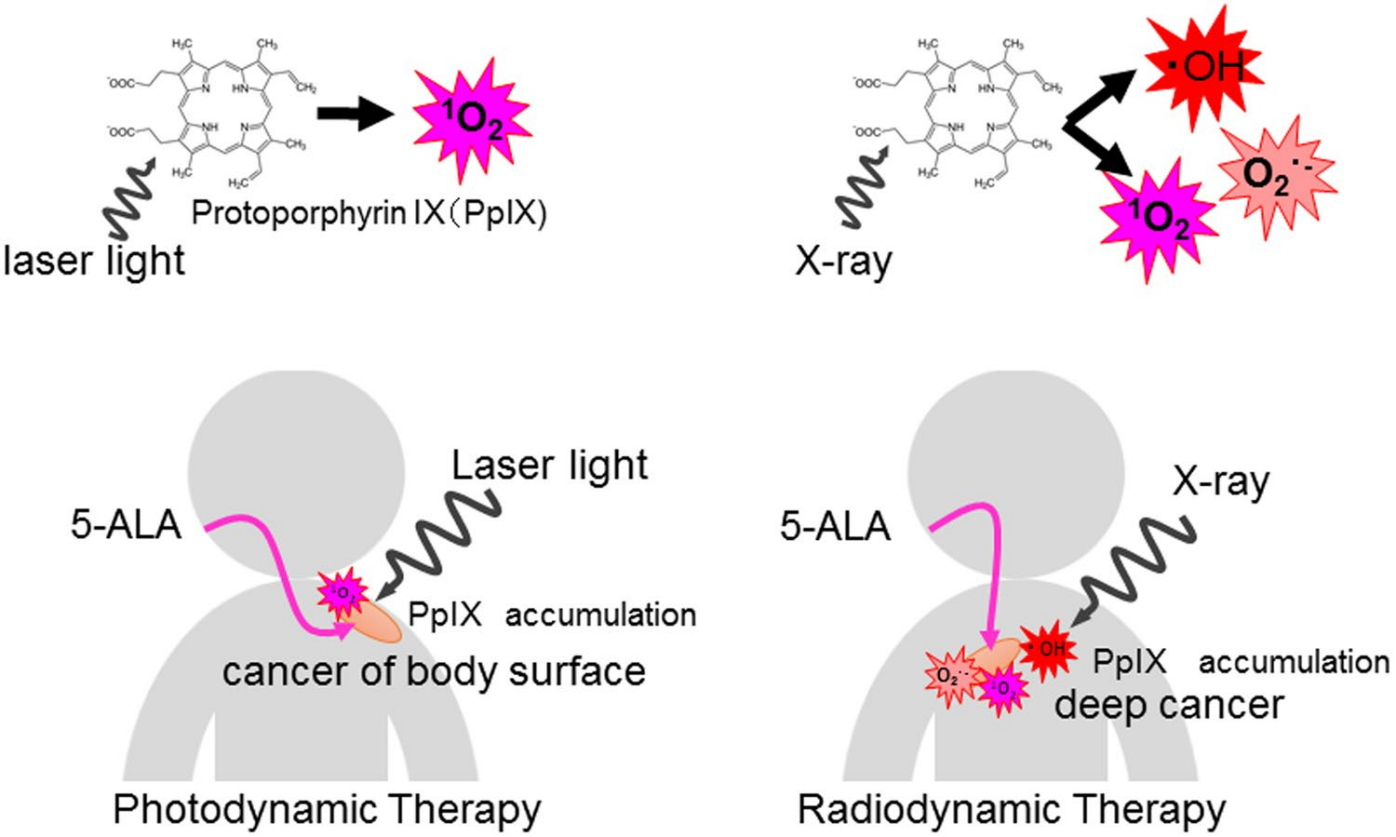
Schematic illustration of radiodynamic therapy. PpIX contributes towards the enhanced generation of ∙OH, O_2_^∙−^, and ^1^O_2_ in the presence of X-ray irradiation. ^1^O_2_ is thought to be a major ROS produced by photodynamic therapy.

The mechanism of tumor suppression by combined treatment with X-ray and 5-ALA is not fully understood. Excess exogenous 5-ALA leads to a build-up of PpIX, which accumulates selectively in epithelial tissues and tumors^9^. PpIX not only contributes to enhanced generation of ∙OH in the presence of X-ray irradiation but also O_2_^∙−^ and ^1^O_2_ by physicochemical reactions^6^. ROS generated by PpIX accumulating in tumor cells caused by X-ray irradiation caused cell cycle arrest^14^. 5-ALA is thus thought to improve the efficacy of cancer radiotherapy by acting as a radiomediator^11^. Although the reactive oxygen species are different, this method, named “radiodynamic therapy”, used radiation as a physical driving force instead of the laser light used in photodynamic therapy (Fig. 6).

Kennedy reported topical application of 5-ALA for sensitization of cutaneous basal cell carcinomas for PDT^21^. Grant showed that oral squamous cell carcinoma can synthesize and accumulate photosensitizing levels of PpIX^22^. For other cancers, such as brain tumors, bladder cancer, and prostate cancer, the accumulation of PpIX following systemic administration of 5-ALA is considered to be a common characteristic of cancer^23^. Because radiation can treat cancer at a greater depth than laser light, radiodynamic therapy could more useful for many additional types of cancer than photodynamic therapy.

Microarrays allow for the detection of genome-wide perturbations during treatment, and the measurement of responses by gene probes. Previously, we examined the effect of combined treatment with X-ray irradiation and 5-ALA using CP-160, which induced changes in the expression of genes related to cell-cycle arrest^14^. In the present study, we achieved similar results using linac (Supplementary Table 2). We also investigated the effect of radiation dose, and found that the gene expression profile of ALA-20XT is located between that of 20XT and 30XT/ALA-30XT according to clustering and correlation analysis (Fig. 3B,C).

The biological effect of radiotherapy is caused by damage from either direct or indirect ionization of the atoms that make up the DNA chain. Indirect ionization occurs because of the ionization of water, forming free radicals, notably ∙OH. PpIX, which accumulates in cell following the administration of 5-ALA, causes the production of ^1^O_2_ or O_2_^∙−^ during X-ray irradiation. GADD45 is a growth arrest and DNA-damage-inducible protein, while P21 is a cyclin dependent kinase inhibitor that is induced following DNA damage. Both *p21(Cdkn1a)* and *Gadd45a* are typically induced by X-ray irradiation. These genes have the largest and most persistent responses to X-ray exposure, and have previously been used for radiation dosimetry by expression analysis in blood from 20 normal healthy human donors^24^. In the present study, the mRNA levels of *p21* and *Gadd45a* increased following X-ray irradiation, regardless of dose or 5-ALA treatment (Fig. 4A,B). The expression of antioxidant enzymes *Sod2* and *Gpx7* increased with increased dose, and with 5-ALA treatment (Fig. 4C,D).

In the *in vitro* cell cycle assay, we observed that 2-Gy X-ray irradiation increased cell cycle arrest at the G2/M phase. This phenomenon increased slightly with 3-Gy irradiation, while the cell cycle arrest increased in both 2-Gy and 3-Gy irradiation with 5-ALA treatment compared to X-ray treatment alone (Supplementary Figure 1). Since the *in vitro* irradiation dose is equal to one day’s dose *in vivo*, the effect of irradiation *in vivo* should be 10 times that of what is seen *in vitro*. Thus, 5-ALA treatment seems to enhance the suppression of tumor growth with X-ray treatment regardless of irradiation dose by enhancing the cell cycle arrest.

The lifetime of ROS is short. Also, it is difficult to operate measuring devices simultaneously with X-ray irradiation. For this reason, detection of ROS generated due to X-rays is extremely difficult. In the present study, the luminescent signals produced by coelenterazine after 3 Gy X-ray irradiation increased and subsequently decreased with time, and became more conspicuous in tumor with 5-ALA treatment (Fig. 5C,D). If the signal is due to ROS, O_2_^∙−^ should be measured^16,17^. Meanwhile, we observed PpIX contributes towards the enhanced generation of ∙OH, O_2_^∙−^, and ^1^O_2_ in the presence of X-ray irradiation^6^. Further studies are required for understanding whether ROS are directly generated by X-rays or generated secondarily, or what biological effect the different kinds of ROS have as a result of 5-ALA treatment.

Melanomas are known to be resistant to radiation. This could be explained by the activation of certain oncogenes, which decreases the sensitivity of cells towards ionizing radiation^25^. Modern techniques, such as stereotactic radiosurgery and stereotactic radiotherapy, are useful for treating high-risk melanomas to prevent locoregional recurrence; however, these techniques have failed to improve the overall survival rate. A number of case reports on the use of radiotherapy combined with targeted therapies, such as the use of BRAF inhibitor or lpilimumab, were published^26,27^. They can increase the radiosensitivity of melanomas cells and normal tissues that can lead to an increased risk of toxicity. On the other hand, 5-ALA, which shows high selectivity for cancer cells with very low toxicity, has been approved for use in high-grade glioma surgery as a precursor of the natural photosensitizer PpIX in PDD and/or PDT in Europe, Japan, and USA^28^. Further *in vivo* studies on all types of melanomas and clinical trials are needed to investigate the usefulness of combined treatment of melanoma with radiotherapy and 5-ALA.

In summary, we have demonstrated that 5-ALA works as a radiosensitizer with X-ray irradiation, using both a high-energy medical linac accelerator as well as a low energy source. 5-ALA treatment improved tumor suppression when combined with X-ray irradiation in the ALA-20XT group regardless of X-ray apparatus. The gene expression profiles of the ALA-20XT group resemble those of 30XT and ALA-30XT along with shrinking of tumor volume. In terms of molecular response, X-ray irradiation seems to cause cell-cycle arrest, and 5-ALA treatment enhanced the tumor suppression at both irradiation doses. 5-ALA has been used clinically for fluorescence-guided surgery and photodynamic therapy. Thus, radiodynamic therapy, which uses radiation as the physical driving force instead of the laser light used in photodynamic therapy, might practical clinical applications. Further studies must first establish practical procedures to optimize the use of radiodynamic therapy with combined 5-ALA treatment.

## Electronic supplementary material

Supplementary table 1

Supplementary table 2

Supplementary figure 1
