# Supplementary material for: Verification of radiodynamic therapy by medical linear accelerator using a mouse melanoma tumor model
Source: Sci Rep. 2018 Feb 9;8:2728. doi: 10.1038/s41598-018-21152-z (PMC5807383; doi:10.1038/s41598-018-21152-z)
Supplement: Supplementary file 1 — Supplementary table 1 [file 41598_2018_21152_MOESM1_ESM.doc]

**Supplementary table 1.** Summary of the microarray data (number of genes)

|  | 20XT | ALA-20XT | 30XT | ALA-30XT |
| --- | --- | --- | --- | --- |
| Up-regulation | 1151 | 863 | 1710 | 1401 |
| Down-regulation | 1272 | 972 | 2199 | 2281 |
| Total | 2423 | 1835 | 3909 | 3682 |

Numver of genes with *p-*values < .01 in the 20XT, ALA-20XT, 30XT and ALA-30XT groups versus NT and ALAT.
