# Supplementary material for: Verification of radiodynamic therapy by medical linear accelerator using a mouse melanoma tumor model
Source: Sci Rep. 2018 Feb 9;8:2728. doi: 10.1038/s41598-018-21152-z (PMC5807383; doi:10.1038/s41598-018-21152-z)
Supplement: Supplementary file 2 — Supplementary table 2 [file 41598_2018_21152_MOESM2_ESM.doc]

**Supplementary table 2.** Differentially represented biological processes based on GO.

|  | | | **Count** | | | | **FDR** | | | | **Gene linst of upregulation or down regulation** | | | |
| --- | --- | --- | --- | --- | --- | --- | --- | --- | --- | --- | --- | --- | --- | --- |
| Function | Accession | Term | 20XT | ALA-20XT | 30XT | ALA-30XT | 20XT | ALA-20XT | 30XT | ALA-30XT | 20XT | ALA-20XT | 30XT | ALA-30XT |
| cell cycle | GO:0000070 | mitotic sister chromatid segregation | 8 |  | 9 | 9 | 1.2.E-03 |  | 6.0.E-03 | 6.1.E-03 | down |  | down | down |
|  | GO:0007049 | cell cycle | 80 | 49 | 118 | 109 | 4.3.E-31 | 7.5.E-13 | 3.2.E-38 | 1.1.E-31 | down | down | down | down |
|  | GO:0007059 | chromosome segregation | 13 |  | 17 | 17 | 4.1.E-03 |  | 5.1.E-03 | 5.2.E-03 | down |  | down | down |
|  | GO:0007067 | mitotic nuclear division | 49 | 26 | 66 | 60 | 2.3.E-23 | 6.6.E-07 | 3.9.E-25 | 2.8.E-20 | down | down | down | down |
|  | GO:0007076 | mitotic chromosome condensation |  |  | 7 |  |  |  | 2.1.E-02 |  |  |  | down |  |
|  | GO:0007094 | mitotic spindle assembly checkpoint |  |  | 8 |  |  |  | 2.3.E-02 |  |  |  | down |  |
|  | GO:0008283 | cell proliferation |  |  | 28 | 28 |  |  | 8.4.E-03 | 8.5.E-03 |  |  | down | down |
|  | GO:0030261 | chromosome condensation |  |  | 8 | 8 |  |  | 5.9.E-03 | 5.9.E-03 |  |  | down | down |
|  | GO:0030308 | negative regulation of cell growth | 16 |  |  |  | 1.3.E-02 |  |  |  | up |  |  |  |
|  | GO:0051301 | cell division | 56 | 30 | 76 | 69 | 1.4.E-23 | 7.9.E-07 | 7.3.E-25 | 9.5.E-20 | down | down | down | down |
|  | GO:0051726 | regulation of cell cycle |  |  |  | 16 |  |  |  | 3.4.E-02 |  |  |  | up |
| DNA metabolic process | GO:0006260 | DNA replication | 34 | 21 | 42 | 45 | 1.1.E-21 | 8.8.E-10 | 2.3.E-21 | 1.6.E-24 | down | down | down | down |
|  | GO:0006261 | DNA-dependent DNA replication | 9 |  |  | 8 | 2.6.E-06 |  |  | 5.9.E-03 | down |  |  | down |
|  | GO:0006270 | DNA replication initiation | 9 |  |  |  | 1.3.E-04 |  |  |  | down |  |  |  |
|  | GO:0006281 | DNA repair | 34 | 22 | 49 | 53 | 7.5.E-09 | 2.8.E-03 | 4.2.E-10 | 1.6.E-12 | down | down | down | down |
|  | GO:0006310 | DNA recombination |  |  | 16 | 20 |  |  | 7.2.E-03 | 6.8.E-06 |  |  | down | down |
|  | GO:0006974 | cellular response to DNA damage stimulus | 36 |  | 54 | 58 | 8.9.E-07 | 5.4.E-01 | 3.9.E-08 | 3.3.E-10 | down |  | down | down |
|  | GO:1900264 | positive regulation of DNA-directed DNA polymerase activity | 6 |  |  | 6 | 8.5.E-04 |  |  | 1.8.E-02 | down |  |  | down |
| RNA metabolic process | GO:0000398 | mRNA splicing, via spliceosome |  |  | 21 | 20 |  |  | 3.8.E-04 | 1.7.E-03 |  |  | down | down |
|  | GO:0006364 | rRNA processing |  |  |  | 19 |  |  |  | 4.3.E-02 |  |  |  | down |
|  | GO:0006397 | mRNA processing | 25 |  | 49 | 46 | 3.5.E-03 |  | 7.7.E-10 | 4.1.E-08 | down |  | down | down |
|  | GO:0008380 | RNA splicing | 21 | 13 | 41 |  | 5.0.E-03 | 4.1.E-08 | 2.2.E-09 | 5.5.E+00 | down | down | down |  |
|  | GO:0051028 | mRNA transport |  |  | 16 |  |  |  | 1.8.E-02 |  |  |  | down |  |
| other | GO:0006629 | lipid metabolic process |  |  |  | 37 |  |  |  | 3.5.E-02 |  |  |  | up |
|  | GO:0006810 | Transport |  |  | 122 |  |  |  | 5.6.E-03 |  |  |  | up |  |
|  | GO:0008152 | metabolic process |  |  | 48 | 44 |  |  | 5.5.E-04 | 1.8.E-02 |  |  | down | down |
|  | GO:0015031 | protein transport |  |  | 51 |  |  |  | 1.8.E-02 |  |  |  | up |  |
|  | GO:0055114 | oxidation-reduction process | 42 |  | 63 | 58 | 4.2.E-02 |  | 8.9.E-05 | 1.0.E-05 | up |  | up | up |

Genes with significantly different expression in each group (20XT, ALA-20XT, 30XT, and ALA-30XT) were identified using the functional annotation chart in the Visualization and Integrated Discovery resource (DAVID Bioinformatic Resources 2007, National Institute of Allergy and Infectious Disease, http://apps1.niaid.nih.gov.dafid). To characterize gene expression, Fisher's exact test was applied to calculate the significance for GO terms related to biological processes. Terms with adjusted *p*-values < .01 (Benjamini-Hochberg FDR correction) were defined as statistically significant.
