# Supplementary material for: Verification of radiodynamic therapy by medical linear accelerator using a mouse melanoma tumor model
Source: Sci Rep. 2018 Feb 9;8:2728. doi: 10.1038/s41598-018-21152-z (PMC5807383; doi:10.1038/s41598-018-21152-z)
Supplement: Supplementary file 3 — Supplementary figure 1 [file 41598_2018_21152_MOESM3_ESM.doc]

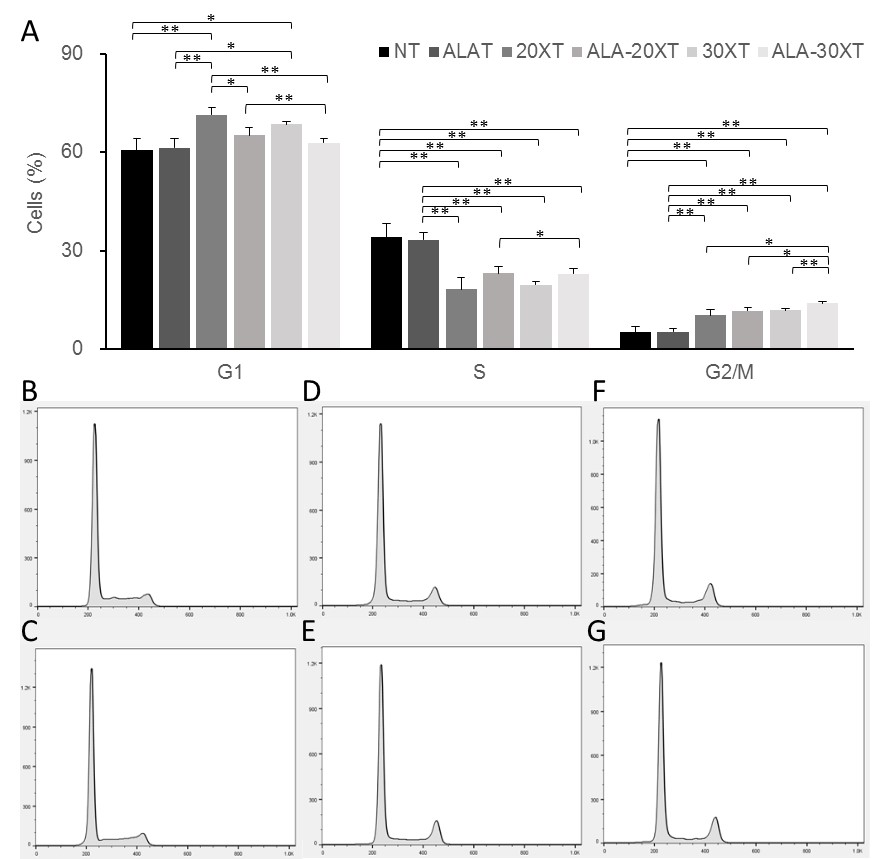


**Supplementary figure 1.** Cell cycle distribution 24 h after 2 or 3 Gy irradiation of B16/Bl6 cells, with or without 24h pre-treatment of 50µg/ml 5-ALA *in vitro*. Cells were subjected to flow cytometry following PI staining (A). Data are the means ± SD (n=6, * *p* < 0.05, ** *p* < 0.01). (A) Representative single parameter histograms of PI fluorescence (DNA content). (B) no treatment, (C) 5-ALA, (D) 2Gy X-ray irradiation, (E) 5-ALA treatment flowed by 2Gy X-ray irradiation, (F) 3Gy X-ray irradiation, and (G) 5-ALA treatment flowed by Gy X-ray irradiation.
